# ﻿*Walsuraguangxiensis* (Meliaceae), a new species from Guangxi, China

**DOI:** 10.3897/phytokeys.234.106205

**Published:** 2023-10-26

**Authors:** You Nong, Chuan-Gui Xu, Gui-Yuan Wei, Ke-Jian Yan, Xin-Cheng Qu, Zhan-Jiang Zhang, Ren-Chuan Hu, Yun-Feng Huang

**Affiliations:** 1 Guangxi Key Laboratory of Traditional Chinese Medicine Quality Standards, Guangxi Institute of Chinese Medicine & Pharmaceutical Science, No. 20-1 Dongge Road, Nanning, Guangxi, China Guangxi Key Laboratory of Traditional Chinese Medicine Quality Standards, Guangxi Institute of Chinese Medicine & Pharmaceutical Science Nanning China; 2 Guangxi Key Laboratory of Medicinal Resources Protection and Genetic Improvement, Guangxi Botanical Garden of Medicinal Plant, Nanning 530023, China Guangxi Key Laboratory of Medicinal Resources Protection and Genetic Improvement, Guangxi Botanical Garden of Medicinal Plant Nanning China

**Keywords:** China, Meliaceae, new species, taxonomy, *Walsuraguangxiensis*

## Abstract

*Walsuraguangxiensis* (Meliaceae), a new species from Guangxi, China, is here described and illustrated. The new species is easily distinguishable from the other two Chinese members of the genus by its petals being pale yellow, filaments being connate into tubes above the middle, the berry being oval and glabrous. An identification key of *Walsura* for 17 species is also provided.

## ﻿Introduction

Researchers have been continuously enhancing the classification of the Meliaceae family through various studies (Muellner et al. 2003, 2005, 2009a, b; Muellner and Mabberley 2008; Pennington and Muellner 2010; Köcke et al. 2015; Clarkson et al. 2016; Gama et al. 2020). Furthermore, new species are still being uncovered and documented (Pannell et al. 2020). *Walsura* Roxb. is a small genus of the family and, according to the International Plant Names Index (IPNI), 53 binomials are referable to *Walsura*, but most of them now considered synonyms of *Walsurapinnata* Hassk. In POWO 16 species are accepted. Clark (1994), in his monography of the genus, recognized 13 species and 3 insufficiently known species, the same as Mabberley in Flora Malesiana (Mabberley et al. 1995). The species of *Walsura* occur in India (west to the Western Ghats and north to Darjeeling), Sri Lanka, the Andaman Islands, Burma, Thailand, Indo-China, Yunnan, Hainan, the Malay Peninsula, Sumatra, Java, Borneo, the northern and western Philippines (Luzon to Palawan), Sulawesi, Halmahera and western New Guinea (Manokwari). Medicinal plants of the genus *Walsura* are native to tropical zones of a number of Asian countries and have been used for local medicines; in addition, the genus has received increasing attention due to its bioactive limonoids and triterpenoids (Son 2022).

Species of the genus grow as trees, sometimes small. Leaves are arranged in spirals, odd-pinnate or occasionally a single leaflet; leaflets are opposite. Flowers are bisexual and male or unisexual (then plants dioecious). The calyx is short, deeply (4)5(6)-lobed, imbricate in bud. Petals 5, much longer than the calyx, distinct, broad and expanding, valvate or imbricate in bud. Stamens 10; filaments flat, broad, usually basally connate into a tube or sometimes discrete, shorter than petals; anthers introrse, inserted on apex or between 2 lobes of filament.

*Walsura* can be distinguished from all other genera in the Meliaceae by its fruit being a berry, indehiscent; its stamen filaments being connate for ± basal half into a staminal tube; its corolla usually being imbricate; loculi uniovulate or with 2 collateral ovules; its anthers inserted apically on filaments or on margin of staminal tube; its disk being annular, fleshy (Pennington and Styles 1975; Clark 1994).

To date, only two species have been recognised in China, *Walsurarobusta* and *Walsurapinnata* (Peng and Mabberley 2008). Very recently, however, eight individuals of a distinctive plant have been found by the first author on limestone in Guangxi, which we describe as a new species below, based on measurements of three individuals.

## ﻿Materials and methods

### ﻿Morphology

The new species is described, based on field observations and examination of herbarium specimens at KUN, PE, IBK, IBSC, GXMI and HITBC. Other *Walsura* species were examined online from the Kew Herbarium (http://apps.kew.org/herbcat/gotoHomePage.do) and Museum national d’histoire naturelle (https://www.mnhn.fr/fr). Morphological characters that distinguish it from all other species in the genus of *Walsura* are used. We observed living plants of the new species at flowering and fruiting time (April to August). We observed characters of stems, leaves, pedicels, flowers, receptacles, petals, stamens, gynoecium, carpels, size of flowers, size and shape of petals, number of stamens and the shape of gynoecium and fruit by studying three individuals.

Descriptions were written based on herbarium specimens. Measurements were made with a tape measure and callipers. The structure of the indumentum and its distribution were observed and described under a dissecting microscope at magnifications of more than 20×. Additional information on locality, habitat, ecology, plant form, bark and wood characters and fruits was collected in the field and taken from herbarium labels. The conservation threat assessment followed IUCN Categories and Criteria (IUCN 2022).

## ﻿Results and discussion

### ﻿Taxonomy

#### ﻿ Walsura guangxiensis

Taxon classificationPlantaeSapindalesMeliaceae

Y.Nong & Y.F. Huang sp. nov.

D39B367E-23EF-5984-99A8-832FF44CEC24

urn:lsid:ipni.org:names:77329399-1

**Figure 1.**
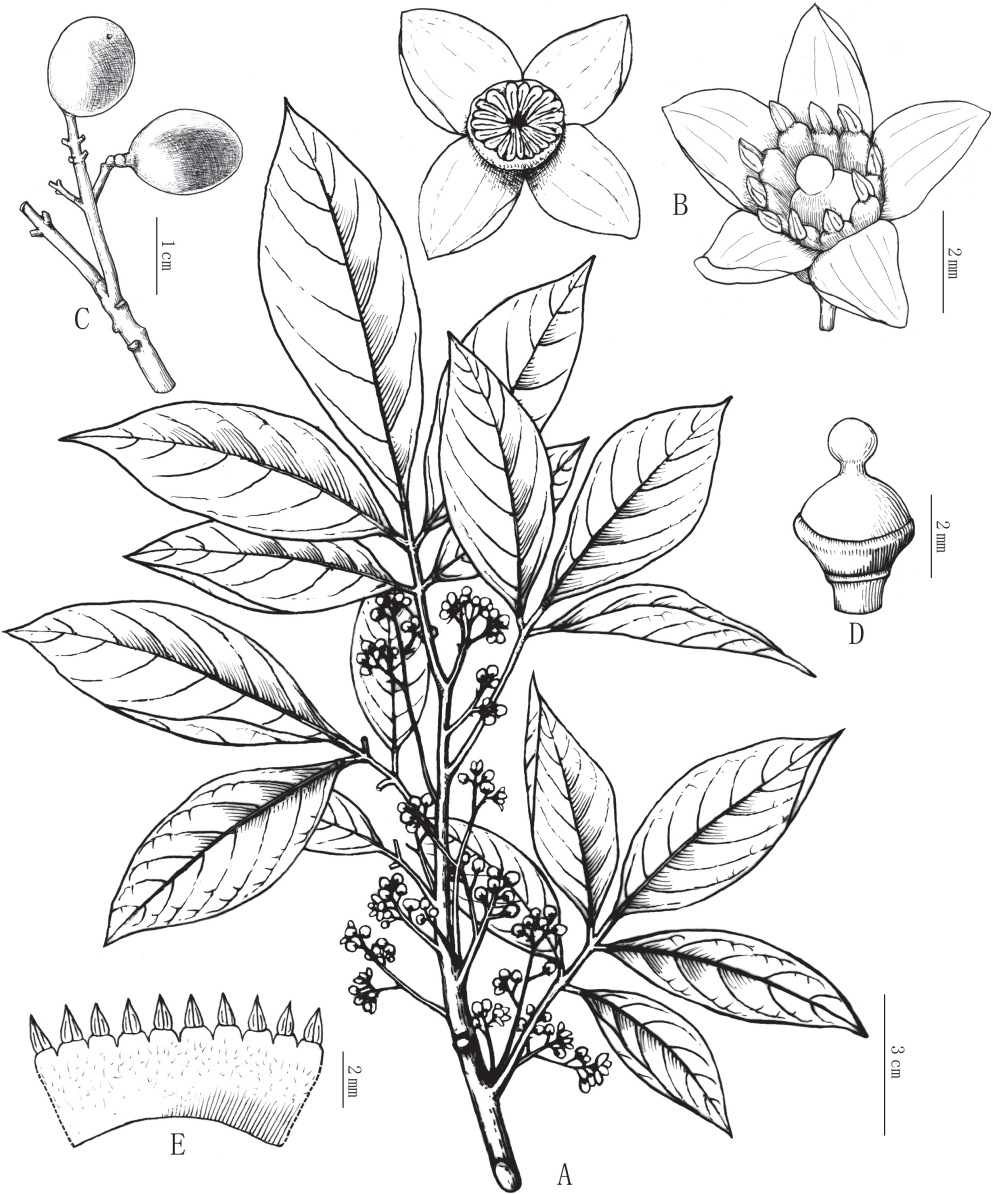
Line drawing of *Walsuraguangxiensis***A** flowering branch **B** flowers **C** fruits **D** style and ovary **E** staminal tube spread out **F** free portion of stamen (Drawn by Xin-Cheng Qu).

**Figure 2.**
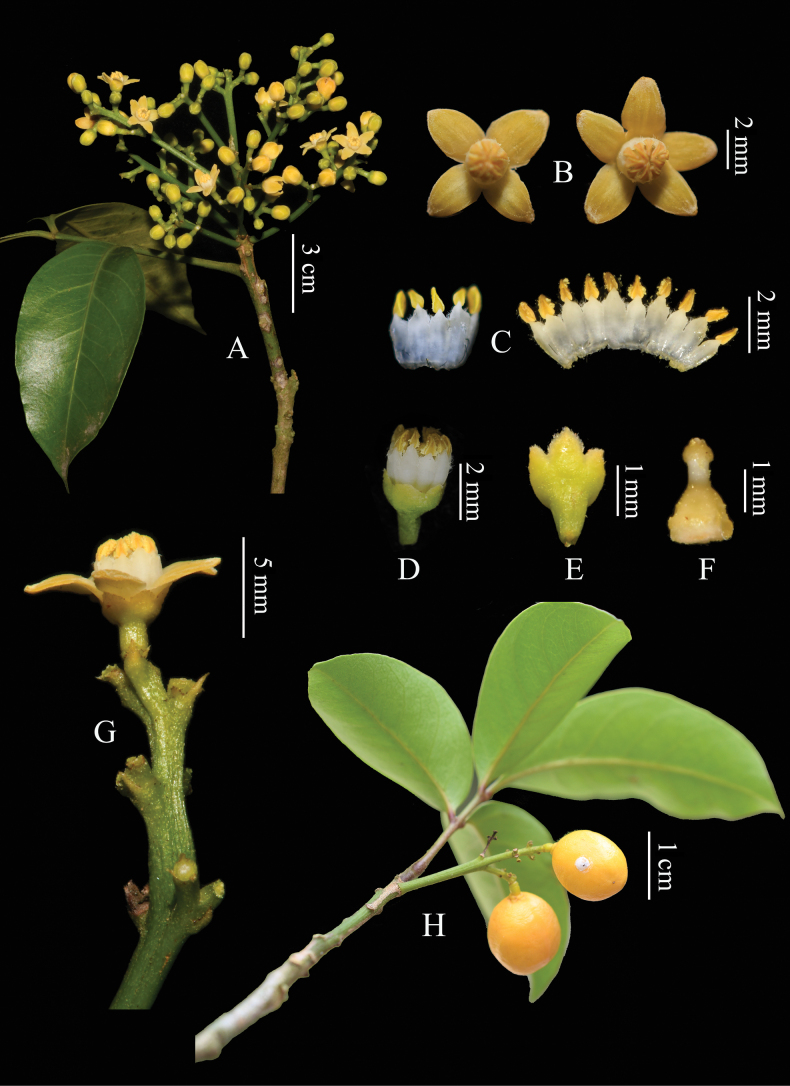
*Walsuraguangxiensis***A** flowering branch **B** flowers **C** staminal filaments **D** staminal tube **E** calyx **F** ovary and stigma **G** flower and stipules **H** fruits (Photographed by You Nong and Ke-Jian Yan, edited by Yuan Fang).

Figs 1 , 2 Chinese name: guǎng xī gē shé shù (广西割舌树)

##### Diagnosis.

*Walsuraguangxiensis* is readily distinguishable from the other two Chinese species of *Walsura*, *Walsuraguangxiensis* is similar to *W.pinnata* and *W.robusta* regarding secondary veins 3–9 (vs. secondary veins 8–11 / secondary veins 5–8); but differs with petals being pale yellow (vs. petals white / petals white); stamen filaments undivided, connate into tubes above the middle (vs. stamen filaments broad, basal to middle part connate into a tube, tip 2-lobed / stamen filament base or basal to middle part connate into a tube); berry oval, 1–2 cm long and 1–1.2 cm wide, glabrous, thin peel, yellow and shiny when mature (vs. berry globose to ovoid, ca. 1.5 cm in diam., densely covered with yellowish gray trichomes / berry globose to ovoid, 1–2 cm in diam., densely covered with yellowish gray trichomes).

##### Holotype.

China. Guangxi: Fengshan, 24°24'29.02"N, 106°50'23"E, alt. 866 m, in subtropical evergreen broad-leaved forest, limestone, 7 June 2022, *R.C. Hu*, *HRC210424001* (holotype: GXMI!; isotypes:IBK! GXMI!).

##### Description.

Trees 3–5 m tall. Branches grey-brown, glabrous or sometimes young branches yellow pubescent or glabrescent, with grey-white lenticels. Leaves 10–26 cm; petioles (1.5) 3–7 cm; with fine hairs. Leaflets 3 or 5, subsessile, papery or thinly leathery, elliptic or oblong-lanceolate, tapering at the apex, descending at the base or cuneate or broadly cuneate, glabrous on both sides, 3–9 pairs of lateral veins, obvious protrusions on both sides of reticular veins and entire edges of leaflet blades; lateral leaflets are 3–14 cm long and 1.5–5 cm wide and the apical leaflets are larger. Petiole 2–5 mm long, terminal up to 1 cm, glabrous. Panicle axillary or terminal, with cyme-like branches, shorter leaves, appressed yellow pubescence, with total pedicels, small pedicels 1–3 mm long, jointed at the lower part and puberulent, 3 triangular bracteoles at the base, pubescence. Calyx short, 4- or 5-lobed, lobes ovate, apex acute, puberulent or glabrous. Petals 4 or 5, pale yellow, puberulent outside; elliptical, much longer than sepals, free, imbricate in bud. Stamen filament tube 8–10 split; filaments are tapered at the top, undivided, connate into tubes above the middle, with short bristles on the upper part of the inner surface and anthers 8–11, yellow and oval, inserted at the top of filaments. Disc cup-shaped, fleshy. Ovary glabrous, 1-loculed, with 2 ovules in each locule, as long as or slightly longer than the ovary. Style cylindrical; stigma globose, tip not divergent. Berry is oval, stipitate, 1–2 cm long and 1–1.2 cm wide, glabrous, 1 loculed, with 1 or 2 seeds, thin peel, yellow and shiny when mature.

##### Phenology.

Flowering in April-May; fruiting in June-August.

##### Etymology.

Guangxi is located in the southwest of China and is a biodiversity hotspot where many new species or new species records have been found (Hu et al. 2019; Luo et al. 2020; Feng et al. 2021; Xin et al. 2021; Huang et al. 2022). The new species, *W.guangxiensis*, is found in this region and is named after the geographic location.

##### Distribution and habit.

Known only from the southwest of Guangxi, China. The new species mainly occurs at elevations of 800 m and is usually found together with *Cinnamomumsaxatile* H. W. Li, *Myrsinekwangsiensis* (E. Walker) Pipoly & C. Chen, *Platycaryastrobilacea* Sieb. et Zucc., *Wrightiasikkimensis* Gamble. It often grows in stone crevices with barren soil.

##### IUCN Red List Category.

Data available for the new species are still insufficient to assess its conservation status. According to the IUCN Criteria (IUCN 2022), it is considered Data Deficient (DD) until more information becomes available. Although *W.guangxiensis* currently has relatively good growth and protection status, further collection and monitoring are necessary to allow more conclusive estimations about the rarity and vulnerability of the species. Therefore, special attention should be given to the conservation of the new species of *Walsura*.

##### Additional specimen.

Tiane. Southwest Guangxi: limestone hills, fl. 8 May 2020, *C.G. Xu*, *XCG20200508001* (GXMI!);Lingyun. Yuntai Park, fr. 7 June 2013, *GXMI063377* (GXMI!); Lingyun. Yuntai Mountain, fr. 12 August 2013, GXMI063363 (GXMI!)

##### Notes.

This new species is represented by eight individuals that have been found so far in the wild, three of which were fruiting and used for species description.

### ﻿Key to species of *Walsura*

**Table:** 

1	Single leaf	**2**
–	Compound leaf	**3**
2	Peduncle of inflorescence with 2-armed trichomes; androecium tubular for less than 1/6 of length	**1. *W.gardneri***
–	Peduncle of inflorescence with simple trichomes only; androecium tubular for more than 1/3 of length	**2. *W.monophylla***
3	Leaves 1-jugate	**4**
–	Leaves 2-or more-jugate	**6**
4	Leaflets slightly asymmetric; filament apex truncate	**3. *W.bonii***
–	Leaflets symmetric; filament apex shortly bifid	**5**
5	Leaf apex acuminate	**4. *W.tubulata***
–	Leaf apex obtuse	**5. *W.trifoliolata***
6	Leaflet abaxial surface white-dotted (matt/glaucous in islets); stamen filament base or basal to middle part connate into a tube	**6. *W.robusta***
–	Leaflet abaxial surface not white-dotted (matt/glaucous uniformly); stamen filaments basal to middle part connate into a tube or connate into tubes above the middle	**7**
7	Leaflet abaxial surface velutinous	**7. *W.poilanei***
–	Leaflet abaxial surface glabrous to subdensely pubescent	**8**
8	Fruit 4-winged to rhomboid (in transverse section) and weakly dehiscent	**8. *W.dehiscens***
–	Fruit globose and indehiscent	**9**
9	Fruit slightly beaked	**10**
–	Fruit not beaked	**13**
10	Leaflet blades lanceolate	**11**
–	Leaflet blades ovate, ovate-lanceolate or elliptic	**12**
11	Inflorescences axillary, crowded at ends of branches, puberulous; fruits ovoid to globose, ca 2 cm long, minutely rusty-puberulous with a slightly curved conical apiculus	**9. *W.candollei***
–	Inflorescences clustered around shoot apex in axils of caducous undeveloped or fully expanded leaves, primary rachis minutely pubescent, branches and all other parts densely puberulous; fruits ovate-oblong, acuminate, ca 1.2 cm long, greyish-velvety	**10. *W.oxycarpa***
12	Inflorescences subcorymbose cymes, axes densely fulvous pilose; fruits ellipsoid, 2.5–3 cm long, brownish green tomentellous, apex usually apiculate	**11. *W.decipiens***
–	Inflorescence panicle, pubescent; fruits ovoid to globose, 1.5–2 cm long, brownish yellow tomentellous, apex usually apiculate	**12. *W.trichostemon***
13	Leaves 2- (or 3-)jugate	**14**
–	Leaves 3- (or 4-) jugate	**16**
14	Leaflet apex obtuse	**13. *W.villosa***
–	Leaflet apex acute or acuminate	**15**
15	Petals white, filaments of stamens 2-toothed at the top; berry globose to ovoid, ca. 1.5 cm in diam., densely covered with yellowish gray trichomes	**14. *W.pinnata***
–	Petals yellow, filaments of stamens not divided at the top; berry oval, 1–2 cm long and 1–1.2 cm wide, glabrous	**15. *W.guangxiensis***
16	Leafy twigs 2.5–8.0 mm diam.; leaves 3-jugate	**16. *W.sarawakensis***
–	Leafy twigs 8–15 mm diam.; leaves 4- (or 5-) jugate	**17. *W.pachycaulon***

### ﻿Discussion

To date, in total, there are 17 species accepted in *Walsura*. Amongst these 17 species, *Walsuraguangxiensis* is unusual in the genus with its petals being yellow, filaments not divided at the top, and the berry being oval, stipitate, 1–2 cm long and 1–1.2 cm wide, glabrous, 1-loculed, with 1 or 2 seeds, thin peel, yellow and shiny when mature.

## Supplementary Material

XML Treatment for Walsura guangxiensis

## ﻿Additional information

### Author ORCIDs

You Nong https://orcid.org/0000-0001-7004-0946

Chuan-Gui Xu https://orcid.org/0009-0000-6263-3821

Gui-Yuan Wei https://orcid.org/0000-0003-0652-1213

Ke-Jian Yan https://orcid.org/0000-0002-4927-4665

Xin-Cheng Qu https://orcid.org/0009-0009-9078-9976

Ren-Chuan Hu https://orcid.org/0000-0002-0941-7203

### Data availability

All of the data that support the findings of this study are available in the main text.
